# Supplementary material for: Exosomes derived from HIV-1-infected cells promote growth and progression of cancer via HIV TAR RNA
Source: Nat Commun. 2018 Nov 2;9:4585. doi: 10.1038/s41467-018-07006-2 (PMC6214989; doi:10.1038/s41467-018-07006-2)
Supplement: Supplementary file 1 — Supplementary Information [file 41467_2018_7006_MOESM1_ESM.pdf]

Supplementary Information

Exosomes derived from HIV-1-infected cells promote growth and progression of cancer via HIV TAR RNA

Chen, et al.

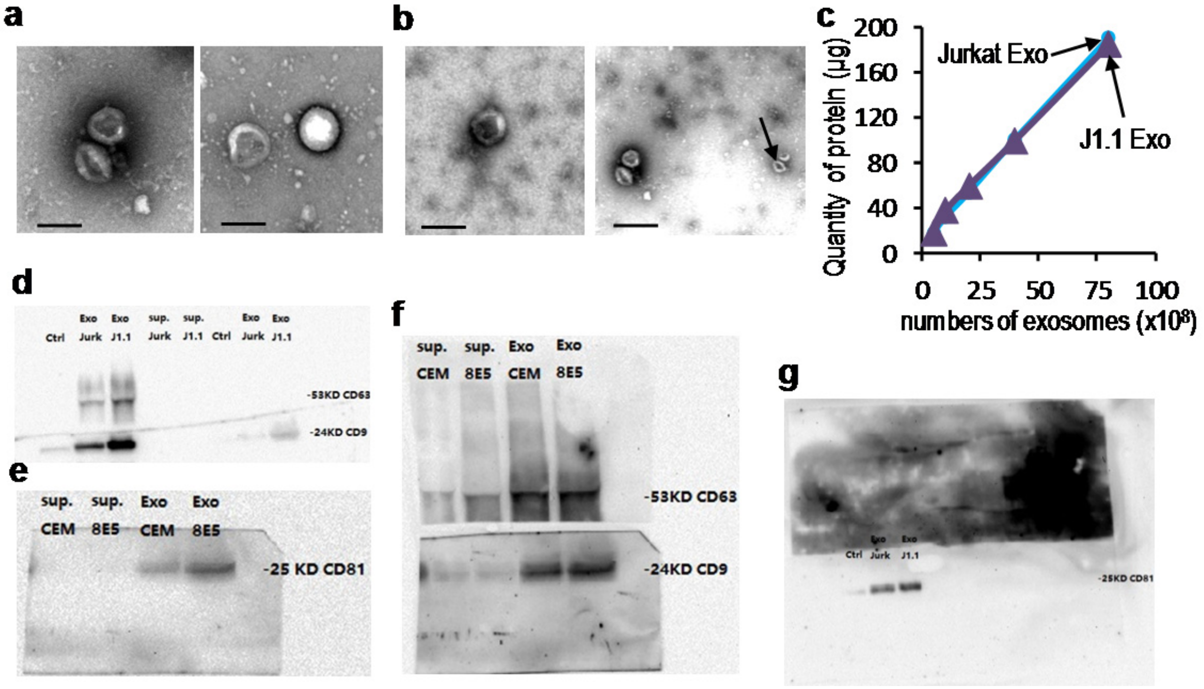

Supplementary Figure 1. Transmission electron microcopy of exosomes purified from Jurkat cell culture supernatants (**a**) and human plasma (**b**). Arrow, plasma exosomes sized around 30 nm. Scale bars, 100nm. (**c**) Total protein quantification on exosomes isolated from Jurkat and J1.1 cells. Numbers of exosomes were determined by the EXOCET exosome quantification assay (System Biol Inc., Palo Alto, CA). Whole immunoblot images of (**d**) CD63 and CD9 on exosomes purified from culture supernatants (Exo) of Jurkat and J1.1 as well as culture supernatants (sup.) of the cells, (**e**) CD81 on CEM and 8E5/LAV exosomes isolated from culture supernatants (Exo) and supernatant controls (sup.), (**f**) CD63 and CD9 on exosomes isolated from CEM and 8E5/LAV culture media (Exo) and supernatant controls (sup.), and (**g**) CD81 on exosomes isolated from culture media of Jurkat and J1.1 cells (Exo).

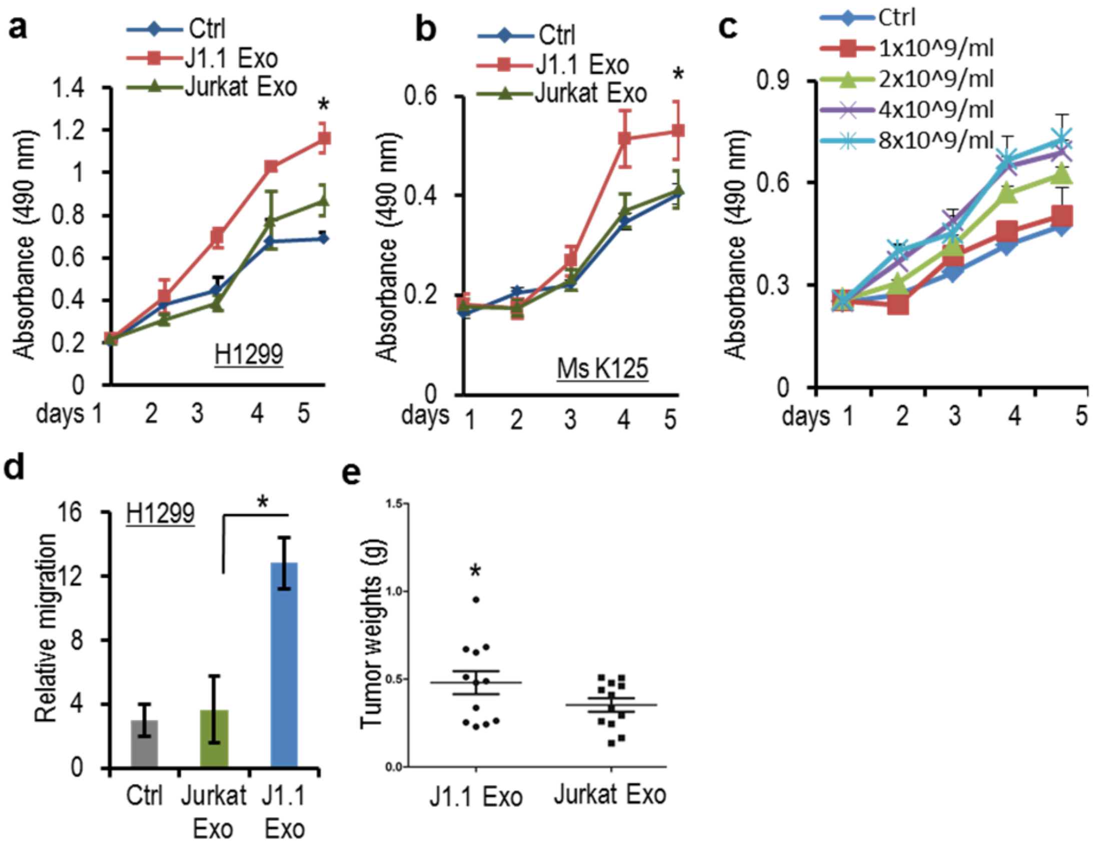

Supplementary Figure 2. J1.1 cell exosomes promote HSC3 HNSCC cells (Dose-response) and lung cancer cell proliferation and migration. (**a**) HIV-1-infected J1.1 cells exosomes enhanced proliferation of human lung cancer H1299 cells and mouse lung cancer K125 cells. Cells were treated with 4 x10^9 exosomes ml^{-1}. Ctrl, serum-free medium; Exo, exosome treatment. Data (mean ± s.d., n=3) represent one experiment out of three repeats. * p<0.05, *F*-test. (**b**) Migration of H1299 cells in response to J1.1 or Jurkat exosomes at 4 x 10^9 exosomes ml^{-1} using wound-healing assays. Data (mean ± s.d., n=4) represent one experiment out of 4 repeats. * p<0.02, *F*-test. (**c**) Dose-response of HSC3 cell proliferation in response to J1.1 cell exosomes. (**d**) H1299 lung cancer cell migration in response to exosomes from J1.1 or Jurkat cells. Data (means ± s.d., n = 4) represent three independent experiments. *p <0.02, F-test. (**e**) H1299 human lung cancer cells were co-inoculated with J1.1 or Jurkat exosomes into nude mice. Tumors were harvested and weighted 15 days after inoculation. n=12; * p<0.05, Student's *t* test.

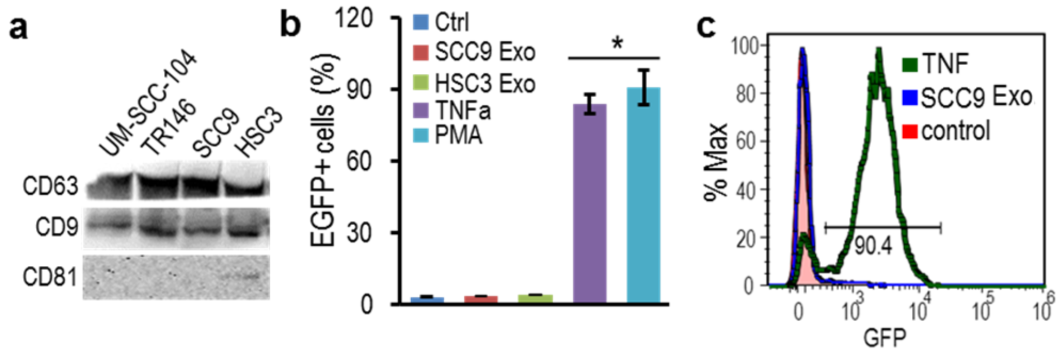

Supplementary Figure 3. Exosomes isolated from cell culture supernatants of HNSCC cells did not reactivate HIV. (**a**) Immunoblots of CD63, CD9 and CD81 in exosomes isolated from NSCC cell culture media. (**b**) 2D10 cells were treated with exosomes from SCC9 and HSC3 cells at 4×10^9 exosomes ml^{-1}, respectively. GFP-positive cells were quantified using flow cytometry and presented as GFP-positive cells/total cells in percentage. 2D10 cells treated with TNFα (5 ng ml^{-1}, TNFa) or PMA (1 nM) for 24 h were used as positive controls. Date (mean ± s.d., n=3) represent one experiment. * $p<0.01$, *F*-test. The experiment was repeated 3 times. (**c**) Flow cytometry of the effect of exosomes from SCC9 cells on HIV activation in 2D10 cells. TNF, TNFα (5 ng ml^{-1}) treatment.

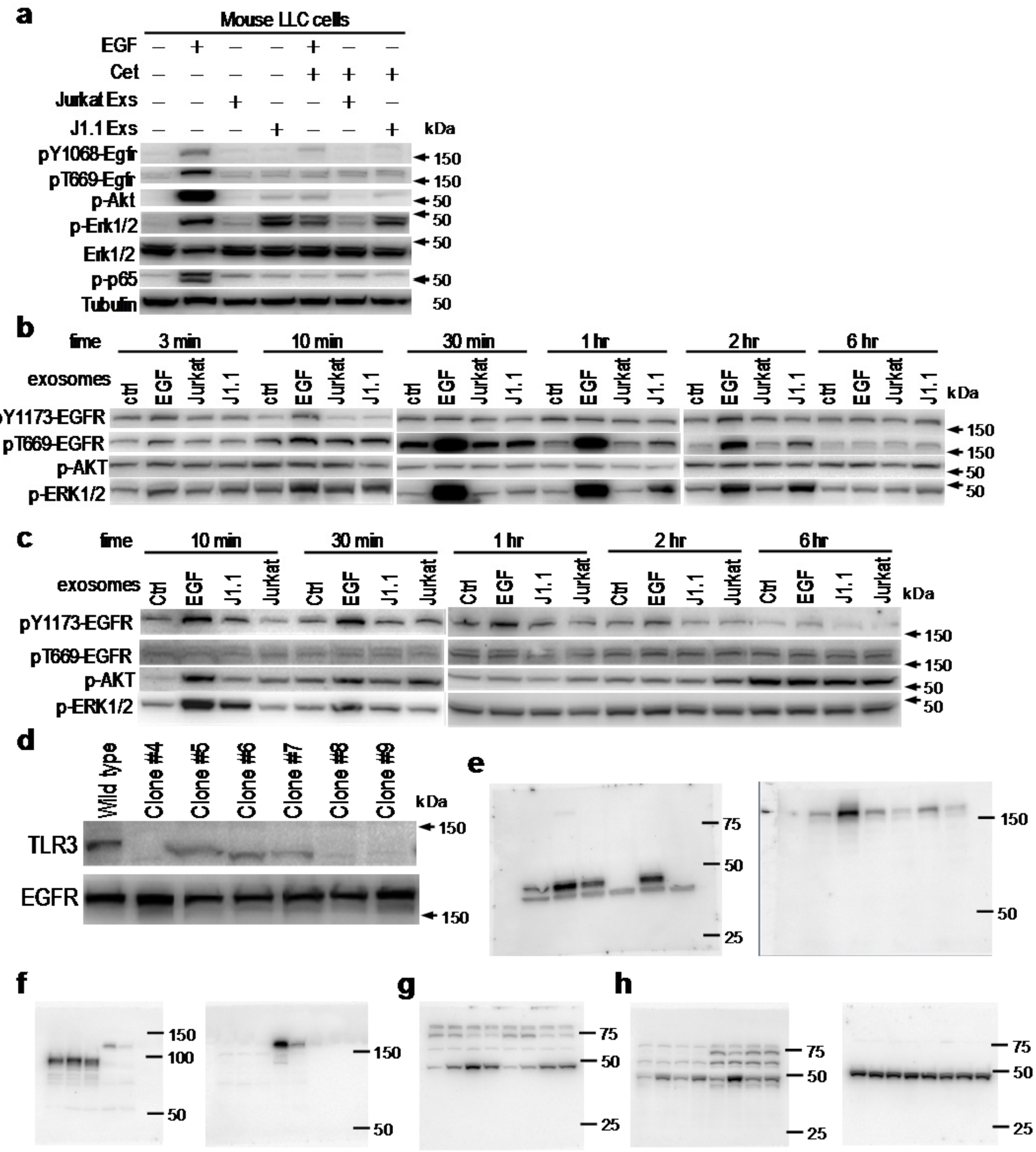

Supplementary Figure 4. EGFR/ERK1/2 signaling in cancer cells treated with exosomes from culture supernatants of J1.1 and Jurkat cell. (**a**) Mouse Lewis lung cancer (LLC) cells were treated with EGF and exosomes from J1.1 or Jurkat cells for 10 min for immunoblotting with antibodies against respective mouse antigens. (**b**) and (**c**) Time-course of ERK1/2 phosphorylation of HSC3 and H1299 cells treated with EGF, J1.1 and Jurkat exosomes, respectively. (**d**) Immunoblots of TLR3 and EGFR on total cell lysates extracted from clones of TLR3 knockout cell lines. Whole immunoblot images of (**e**) phospho-ERK1/2 (left) and phospho-Y1173 (right) of HSC3 cells for Figure 7c, (**f**) CD19 (left) and total EGFR (right) of SU-DHL-16, Pfeiffer, Toledo and H1299 cells for Figure 7e, (**g**) phospho-ERK1/2 of HSC3 cells for Figure 8b and (**h**) phospho-ERK1/2 (left) and total ERK1/2 (right) of wild-type and TLR-KO HSC3 cells for Figure 8c.

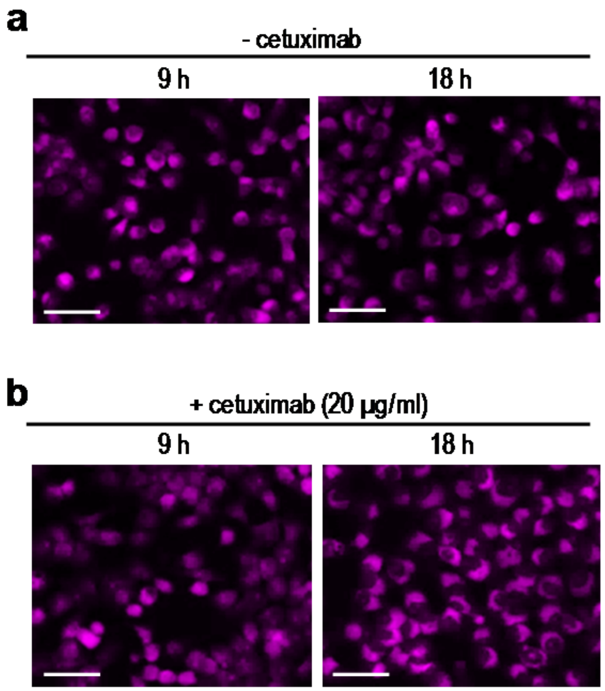

Supplementary Figure 5. Exosome entry into recipient cells. HSC3 cells were added with fluorescently labeled J1.1 cell exosomes for 9 and 18 h in the absence (**a**) and presence of cetuximab (**b**). Exosome entry was visualized by the fluorescence microscope. Scale bars, 100µm.

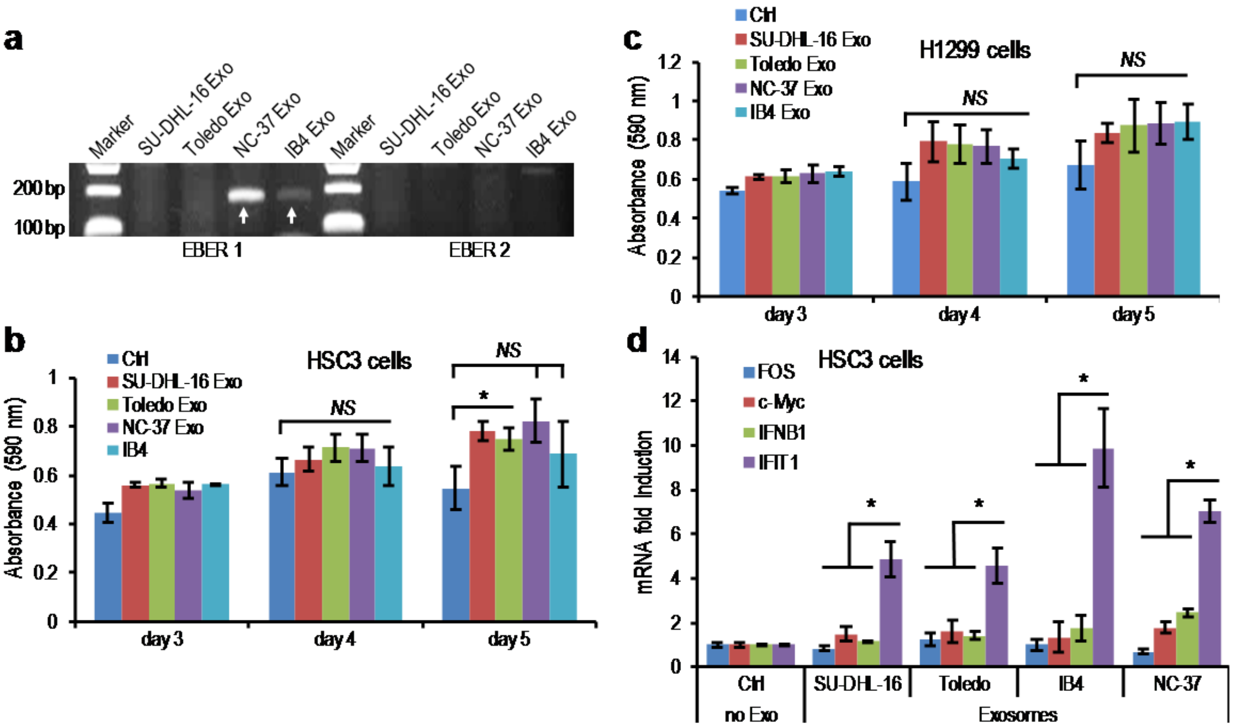

Supplementary Figure 6. Effect of exosomes from EBV+ and EVB- cell lines on proliferation and gene expression of HSC3 cancer cells. (**a**) Gel images of RT-PCR of EBER1 and EBER2 RNA on total RNA extracted from exosomes purified from culture supernatants of EBV+ NC-37 and IB4 cells as well as EBV- SU-DHL-16 and Toledo cells. Exosomes from EBV+ and EBV- cells did not promote proliferation of HSC3 (**b**) and H1299 (**c**) cancer cells. Cancer cells were cultured in the presence and absence of exosomes (4.2 x 10^9 ml^{-1}) from NC-37, IB4, SU-DHL-16 and Toledo cell lines, respectively, and cell proliferation was determined by MTT assays on each day from day 3 to 5. Data represent one assay (n=3) with 2 biological repeats. *NS*, not significant, one-way ANOVA. (**d**) qRT-PCR for *FOS*, c-Myc, *IFNB1* and *IFIT1* mRNA on total RNA extracted from HSC3 cancer cells treated with exosomes from SU-DHL-16, Toledo, IB4 and NC-37 cells for 18 h. Ctrl, no exosome treatment. Data represent one experiment (n=3) out of 2 independent repeats. *, p<0.05, F-test.

Supplementary Table 1. Parameters of HIV-positive and -negative individuals for plasma specimens

Patient ID	Age	Gender	CD4^{+} count	Viral load	ART	Patient ID	Age	Gender
Pt 3827	54	M	258	23	Y	Ctrl 4574	32	F
Pt 1279	67	M	1016	20	Y	Ctrl 4588	50	F
Pt 4011	32	F	692	20	Y	Ctrl 4594	61	F
Pt 1310	52	F	918	20	Y	Ctrl 5075	49	F
Pt 5732	71	M	248	30	Y	Ctrl 6128	41	F
Pt 5332	64	M	440	20	Y	Ctrl 4608	32	F

ART, antiretroviral therapy; Pt, HIV-positive patients; Ctrl, HIV-negative individuals

Supplementary Table 2. Parameters of HIV-positive oral cancer patients

Patient ID	Age	Gender	CD4^{+} count	Viral load	ART	Dx	FFPE	Plasma
Pt 05110	49	M	324	NA	Y	SCC	Y	N
Pt 58460	55	M	651	1180	Y	SCC	Y	Y
Pt 58702	44	M	80	<50	Y	SCC	Y	Y
Pt 58848	56	M	3321	203	Y	SCC	Y	Y
Pt 59802	55	F	66	19356	Y	SCC	N	Y
Pt 59830	54	M	516	<48	Y	SCC	Y	Y

ART, antiretroviral therapy; Dx, diagnosis; SCC, squamous cell carcinoma.

Supplementary Table 3. Concentrations of purified plasma exosomes and exosomes quantified using AChE in unmodified plasma in the same HIV+ and HIV- individuals

subjects	purified exosomes in DMEM ($x10^{11} ml^{-1}$)[a,b]	primary plasma exosomes ($x10^{11} ml^{-1}$)[b]	exo-depl plasma ($x10^{11} ml^{-1}$)[c]	plasma exosome recovery (%)[d]
Pt1114	8.65	7.31	ND	29.6%
Pt2003	6.2	6.14	ND	25.2%
Pt3066	7.69	7.31	ND	26.3%
Pt4520	9.28	6.83	ND	34.0%
Pt4724	8.59	6.09	ND	35.3%
Pt5293	6.59	7.17	ND	23.0%
Ctrl4574	4.75	5.41	ND	22.0%
Ctrl4901	7.15	6.68	ND	26.8%
Ctrl5075	9.2	7.59	ND	30.3%
Ctrl5591	9.55	5.36	ND	44.5%
Ctrl6128	8.1	7.65	ND	26.5%
Ctrl6157	9.28	5.12	ND	45.3%

[a] plasma exosomes were purified from 2 ml plasma of HIV+ patients (Pt) or HIV- individuals (Ctrl) and then resuspended in 0.5 ml of DMEM. [b] exosome concentrations were determined by numbers of exosomes ml^{-1} based on AChE assays. Since the plasma may contain AChE activity, the primary plasma exosomes will likely be overestimated. [c] ND, not determined due to low AChE activity. [d] recovery of purified plasma exosomes was determined by total numbers of purified exosomes in 2 ml of plasma divided by numbers of exosomes in 2 ml of primary plasma from the same person (%).

Supplementary Table 4. Primers used in this report

Gene names	Name/forward primer squence (5'→ 3')	Name/reverse primer sequence(5'→ 3')
HIV Tar	TARfll.F/GGTCTCTCTGGTTAGACC	TARfll.R/GTGGGTTCCCTAGTTAGC
HIV Env	Env.F2019 /GGCAAGTCTGTGGAATTGG	Env.R2187/TGGGATAAGGGTCTGAAACG
HIV Nef	Nef.F31/ATTGGATGGCCTGCTGTAAG	Nef.R206/GGAAAACCCACCTCTTCCTC
HIV Tat	TAT.F33/GAAGCATCCAGGAAGTCAGC	TAT.R225/GGAGGTGGGTTGCTTTGATA
EBER1	EBER1.F/AGGACCTACGCTGCCCTAGA	EBER1.R/ATTCAGTTGAGAAAACATGCGG
EBER2	EBER2.F/AGGACAGCCGTTGCCCTAGTGGTTC	EBER2.R/ATTCAGTTGAGAAAAATAGCGG
DEFB103	hBD3.F5/CTTCTGTTTGCTTTGCTC	hBD3.R3/TTCTTCGGCAGCATTTTCG
IFNB1	IFNB1.F/CGCCGCATTGACCATCTA	IFNB1.R/GACATTAGCCAGGAGGTTCT
IFIT1	IFIT1.F/TCTCAGAGGAGCCTGGCTAAG	IFIT1.R/GTCACCAGACTCCTCACATTTGC
c-Myc	Myc.F1/CCTACCCTCTCAACGACAGC	Myc.R1/CTCTGACCTTTTGCCAGGAG
FOS	FOS.F/ATGGGCTCGCCTGTCAACGC	FOS.R/GGAGATAACTGTTCCACCTTGCCCC
GAPDH (Ms.)	Ms.GAPDH.F1/TGTGTCCGTCGTGGATCTGA	Ms.GAPDH.R1/CCTGCTTCACCACCTTCTTGA
GAPDH (Hu.)	GAPDH.F/TCGGAGTCAACGGATTT	GAPDH.R/CCACGACGTACTCAGC
