# Supplementary material for: Exosomes derived from HIV-1-infected cells promote growth and progression of cancer via HIV TAR RNA
Source: Nat Commun. 2018 Nov 2;9:4585. doi: 10.1038/s41467-018-07006-2 (PMC6214989; doi:10.1038/s41467-018-07006-2)
Supplement: Supplementary file 4 — Description of Additional Supplementary Files [file 41467_2018_7006_MOESM4_ESM.docx]

**Title: Supplementary Movie 1.** **EGFR mediates entry of J1.1 cell exosomes into recipient cells.**
**Description:** Green fluorescent protein (GFP)-tagged EGFR expressing HEK293 cells were incubated in the absence or presence of cetuximab (20 µg/ml) for 30 min, followed by addition of fluorescently labeled J1.1 cell exosomes (red) to the cells. Exosomes were fluorescently labeled using the Exo-Red Exosome Labeling kit following the manufacturer’s instructions (SBI, Palo Alta, CA). The Deltavision RT epifluorescent microscope system was used for imaging. The time-lapse POL (TRANS, to show structure of cells) and red fluorescent (to show exosomes) images in z-series were captured simultaneously. 3-D volume projections were generated using the software and avi movies were exported and converted to mov format.
